# A 7-year-old boy with renal insufficiency and proteinuria after stem cell transplant for T-cell acute lymphoblastic leukemia 

**DOI:** 10.5414/CN107767

**Published:** 2013-02-08

**Authors:** Julie E. Goodwin, Matthew Palmer, Farzana Pashankar, Alda Tufro, Gilbert Moeckel

**Affiliations:** 1 Department of Pediatrics and; 2 Department of Pathology, Yale University School of Medicine, New Haven, CT, USA

**Keywords:** hematopoietic stem cell transplant, chronic kidney disease, graft-vs.-host disease, proteinuria, kidney biopsy

## Abstract

Chronic kidney disease is common in pediatric patients following hematopoietic stem cell transplant. Its etiology is likely multifactorial and depends both on pre-conditioning regimens as well as immunosuppressive therapy and post-transplant prophylactic medications. Graft-vs.-host disease (GVHD) is a common sequela of hematopoietic stem cell transplant and has been associated with the nephrotic syndrome (NS). Here we report a case of a pediatric patient who developed proteinuria and renal insufficiency after stem cell transplant. A kidney biopsy showed chronic interstitial nephritis and extensive foot process effacement, which are likely sequelae of GVHD. Moreover we show decreased CD4 and CD3 lymphocyte counts in the interstitial infiltrate, suggesting that abnormal lymphocyte response might play a role in podocyte injury following GVHD. This case illustrates the importance of the kidney biopsy in the assessment of stem cell transplant-mediated renal failure.

## Introduction 

Chronic kidney disease (CKD) is common in pediatric patients following hematopoietic stem cell transplant and its etiology is multifactorial [1, 2, 3]. Graft-vs.-host disease (GVHD) is a sequela of hematopoietic stem cell transplant and may manifest as proteinuria and nephrotic syndrome (NS) [4]. We present a case of GVHD and proteinuria in a pediatric patient following stem cell transplant which highlights the potential role of T-cells in the pathogenesis of this condition. 

## Case report 

### Clinical history and initial laboratory results 

A 7-year-old boy with a past history of T-cell ALL presented ~ 3 months after 5/6 matched allogeneic hematopoietic stem cell transplant for evaluation of increasing serum creatinine levels. His initial presentation had included a high white count of 133,000/µL with 71% blasts and mild anemia. Peripheral blood flow cytometry showed T-cell ALL, which was confirmed by bone marrow biopsy. There was no initial testicular or CNS involvement. Induction chemotherapy included treatment with cytarabine, vincristine, daunorubicin, prednisone, and methotrexate. Risk assessment, performed after completion of induction therapy placed this patient in the high-risk group, as he had minimal residual disease (MRD). He proceeded directly to consolidation therapy without waiting for count recovery and he received cranial radiotherapy during the delayed intensification phase of treatment. Maintenance chemotherapy was begun ~ 7 months after initial presentation and was complicated by several episodes of fever and neutropenia, acute appendicitis requiring laparoscopic appendectomy and a bone marrow relapse of T-cell ALL. 

As per protocol, the patient began intensive reinduction chemotherapy for children with a bone marrow relapse after standard induction therapy. He received the following agents: vincristine, doxorubicin, prednisone, ifosfamide, etoposide, clofarabine, cytoxan, and nelarabine. After ~ 1 month of therapy, preparations commenced for stem cell transplant. Total body irradiation was a component of his pre-transplant conditioning as was high-dose cytarabine and clofarabine. 14 months after initial presentation the patient received a 5/6 matched stem cell transplant. His post-transplant course was complicated by a central nervous system event that occurred ~ 2 weeks after transplant which resembled HUS/TTP and resulted in deafness and paraplegia.He received GVHD prophylaxis with mycophenolate mofetil (MMF) and tacrolimus. 

Immediately following stem cell transplant the serum creatinine was normal at 0.7 mg/dL and rose gradually over a period of 6 – 8 weeks peaking at 2.5 mg/dL. Concurrently, the patient began to gain weight and develop significant total body edema. His blood pressure remained normal. He did not complain of any diarrhea or skin lesions though skin GVHD was confirmed by biopsy and endoscopy showed grade 1 GVHD of the stomach ~ 60 days after stem cell transplant; these resolved after treatment with several weeks of solumedrol. At the time of the initial nephrology evaluation, post-transplant medications included only physiologic hydrocortisone and mycafungin, a nephrotoxin. He was also maintained on keppra for seizure prophylaxis and nadolol for history of a prolonged QTc thought secondary to cardiac toxicity from preparative regimens. 

A renal ultrasound showed normal size kidneys with slightly increased echogenicity and mild pelvicalyceal dilatation. Liver function tests and electrolytes were normal apart from a low phosphorous of 2.7 mg/dL. A phosphate-wasting tubulopathy was confirmed by urine and serum studies, which showed a TRP of 53%. Complement levels were normal with C3 of 203 mg/dL and C4 of 42 mg/dL and an ANA screen was negative at < 1 : 40. Serial protein/creatinine ratios were 3.8 – 10.0 suggesting nephrotic range proteinuria, though the serum albumin was consistently > 3.5 mg/dL. A 24-h urine collection showed 4.7 mg/kg creatinine, presumably so low due to muscle wasting and paralysis, and 14.6 mg/kg protein. There was no hematuria. As a result of this patient’s persistent and moderately impaired renal function as well as his proteinuria, a renal biopsy was pursued since there were some entities in the differential diagnosis which would potentially change management. Differential diagnosis at the time included progressing CKD, GVHD, bone marrow transplant-associated HUS/TTP, radiation nephritis, BK nephropathy, CMV nephritis, calcineurin inhibitor toxicity, and tubulointerstitial nephritis. 

## Results 

### Kidney biopsy 

**Figure 1.**
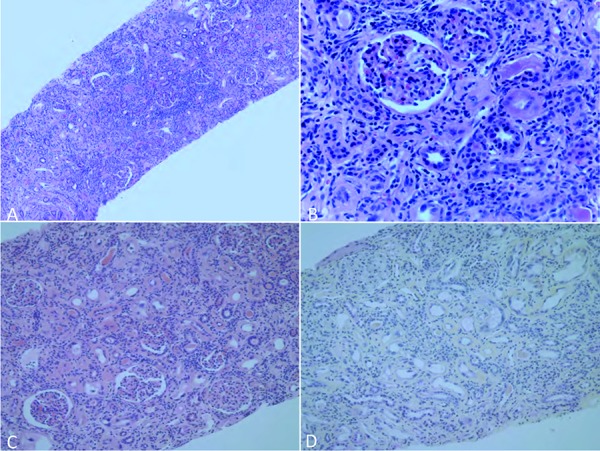
Light microscopy of kidney biopsy shows glomeruli with lymphocytes present within the capillary lumen. Moreover, there was diffuse interstitial fibrosis with proportional tubular atrophy and a predominantly lymphocytic interstitial infiltrate with focal tubulitis (Figure 1 A, H & E 40× and Figure 1B, H & E 200×). Several tubular profiles showed epithelial cells with enlarged nuclei with smudgy chromatin pattern (Figure 1 C, H & E 200× and Figure 1D, HSP 200×).

**Figure 2.**
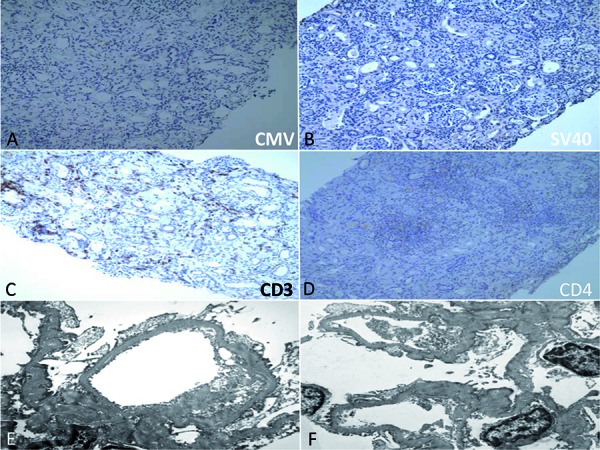
Immunohistochemistry stain for cytomegalovirus (CMV) (Figure 2A, 200×) and SV40 (Figure 2 B, 100×) were negative. Immunohistochemistry stains for CD3 (Figure 2C, 40×) and CD4 (Figure 2D, 100×) showed a predominance of CD3-positive lymphocytes within the interstitial infiltrate (Figure 2C) and only weak, focal CD4 positive lymphocytes (Figure 2D). Electron microscopy studies showed corrugated glomerular basement membranes with extensive and almost complete foot process effacement (Figure 2E, original magnification × 10,000 and Figure 2F, original magnification × 8,000). No immunecomplex deposits were seen.

The kidney biopsy showed two pieces of kidney cortex containing 25 glomeruli. The glomeruli were hypercellular with mildly lobulated tufts and lymphocytes were present within the glomerular capillary lumen. Several glomeruli showed corrugated capillary basement membranes. There was a predominantly lymphocytic interstitial infiltrate, focal tubulitis, and diffuse interstitial fibrosis with proportional tubular atrophy. (Figure 1A, B). Several tubular profiles showed epithelial cells with enlarged nuclei with smudgy chromatin pattern (Figure 1C, D). Immunohistochemistry for cytomegalovirus (CMV) (Figure 2A) and SV40 (Figure 2B) were negative. Immunohistochemistry stains for CD3 and CD4 showed a predominance of CD3 positive lymphocytes within the interstitial infiltrate (Figure 2C) and only weak CD4 positive lymphocytes (Figure 2D). Gomori’s methenamine silver-stained sections were negative for fungi. Immunofluorescence studies showed no specific fluorescence for IgM, IgA, IgG, C3, or C1q. Electron microscopy studies showed corrugated glomerular basement membranes with increased lamina rara interna and extensive, almost complete, foot process effacement (Figure 2E). There were no immune complex deposits present, but there was mild increase in mesangial matrix (Figure 2F). No evidence of thrombotic microangiopathy (TMA) was detected. 

### Diagnosis 

Chronic interstitial nephritis, consistent with GVHD and extensive foot process effacement. 

### Clinical follow-up 

Further work-up was undertaken to determine whether BK nephropathy or anti-phospholipid antibody syndrome may have been contributing factors, given the antecedent history of total body irradiation and the CNS event. An SV40 stain on the kidney biopsy was negative and there was no evidence of a TMA lesion in the kidney. Approximately 2 weeks after the biopsy was performed, the patient was re-admitted to the hospital with a sudden right-sided facial droop. During the evaluation for this symptom he was found to have relapse of his initial ALL by bone marrow biopsy and lumbar puncture. He was started on oral dexamethasone and ~ 1 month later his serum creatinine level had improved to 0.9 mg/dL; however, at this stage of his treatment, the family had elected palliative care only and the patient expired soon after. 

Given the close temporal relationship between this patient’s renal insufficiency and his ALL relapse, the possibility that the lymphocytic infiltrate in the kidney biopsy could represent renal involvement with ALL was entertained. However, the presence of a mixed B- and T-cell infiltrate, without morphologic cell abnormalities indicative of ALL was not supportive of this assumption. Unfortunately, gene rearrangement studies to evaluate the clonality of the lymphocytic infiltrate could not be performed due to lack of sufficient kidney tissue remaining in the specimen. 

## Discussion 

Chronic kidney disease in children following hematopoietic stem cell transplant is fairly common and has been well described in the literature [1, 2, 3]. The etiology of CKD is often multifactorial and includes exposure to total body irradiation, effects of immunosuppressive agents such as cyclosporine, effects of chemotherapeutic agents and other specific nephrotoxic agents, such as amphotericin B and acyclovir, used in post-transplant prophylactic regimens [4]. Primary glomerular diseases including membranous glomerulopathy and minimal change disease leading to nephrotic syndrome, while quite rare, have also been reported in children presenting with renal insufficiency and proteinuria following hematopoietic stem cell transplant [5, 6]. Acute renal failure and chronic kidney disease, following hematopoietic stem cell transplantation, occur in 30 – 50% and 18 – 65% of cases, respectively [7]. Proteinuria after allo-HSCT has been associated with chronic GVHD, irradiation therapy and CMV infection [8, 9, 10]. 

GVHD is an entity that is common and it is estimated that ~ 60% of allograft recipients develop acute GVHD (aGVHD) occurring within the first 3 months after hematopoietic cell transplant [4]. One prospective study found the prevalence of albuminuria and proteinuria at 100 days after transplant to be 64% and 15%, respectively. In this series, albuminuria was associated with aGVHD but not acute kidney injury [11]. Sakoda et al. [12] reported that the onset of NS was accompanied by relapse of GVHD. NS after allo-HSCT is thought to be a manifestation of GVHD [13, 14] and has been associated with decreased CD4+ Tregs, at least in intestinal GVHD lesions [15]. 

A possible relationship between T-helper cells and regulatory T-cells (Tregs) with regard to the pathogenesis of GVHD and proteinuria has been described [16, 17, 18], though the mechanism by which Tregs attenuate proteinuria is unknown. A recent study in allo-HSCT patients that developed GVHD and NS showed decreased CD3 and CD4+ T-cells and elevated serum IFN-γ and TNF-α levels in peripheral blood samples [19]. Our patient’s biopsy showed a significantly decreased level of CD4+ T-cells within the lymphocytic interstitial infiltrate on kidney biopsy, though we do not have serum for T-cell levels. However, available data logically lead to the question of whether decreased intrarenal CD4+ T-cells might be associated with the extensive podocyte injury seen in patients with GVHD and NS [16]. 

Recently, two new models of mouse glomerulonephritis were published which provide further evidence of a link between T-cells and podocyte injury. In the first, ovalbumin-specific CD4+ and CD8+ T-cells were preactivated in vitro and injected into mice. Within 7 days these animals developed a periglomerular mononuclear infiltrate involving T-cells, dendritic cells, and podocyte adhesions to parietal epithelia [20]. These mice went on to develop florid FSGS after prolonged treatment. In the second model, Bao et al. [21] induced severe albuminuria in mice by injection of podocyte-specific sheep immunoglobulins. As in the previous model, after several weeks the animals developed a significant periglomerular infiltrate composed of T-cells, dendritic cells and monocytes/macrophages. While the details of the models are quite specific and not necessarily generalizable, both models showed that binding of antigens to podocytes or expression of antigens by podocytes resulted in glomerular injury that was dependent on antigen-specific CD4+ T-cells. Antigen presentation by dendritic cells or podocytes seems to be a critical step in the intrarenal activation of immune cells that leads to kidney injury [20, 21]. It has been suggested that research directed at understanding the mechanisms of antigen presentation in the kidney and/or the role of podocyte associated antigens in the activation of T-cells may provide therapeutic insight into some forms of human glomerulonephritis [22]. 

In summary, the kidney biopsy findings in our patient show chronic interstitial nephritis, the etiology of which is not entirely clear. However, having eliminated viral and fungal infection, drug-induced AIN and recurrent ALL as potential causes, we favor GVHD as the etiology. This diagnosis is further supported by the presence of extensive foot process effacement and endothelial injury. The scarcity of CD4+ T-cells in the interstitial infiltrate is also suggestive of GVHD and highlights the question of whether patients with GVHD and low CD4+ T-cell count are at increased risk for extensive podocyte injury. 

## Conflict of interest 

The authors have no conflicts of interest to disclose.
